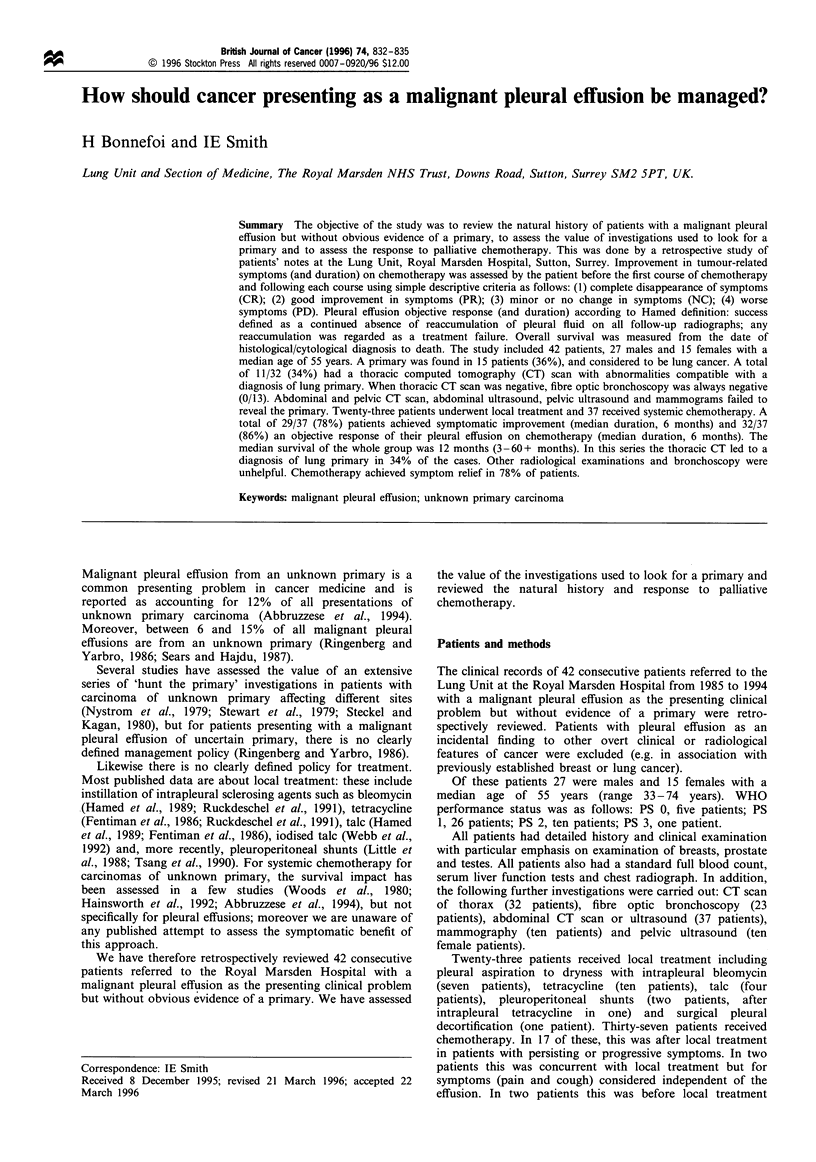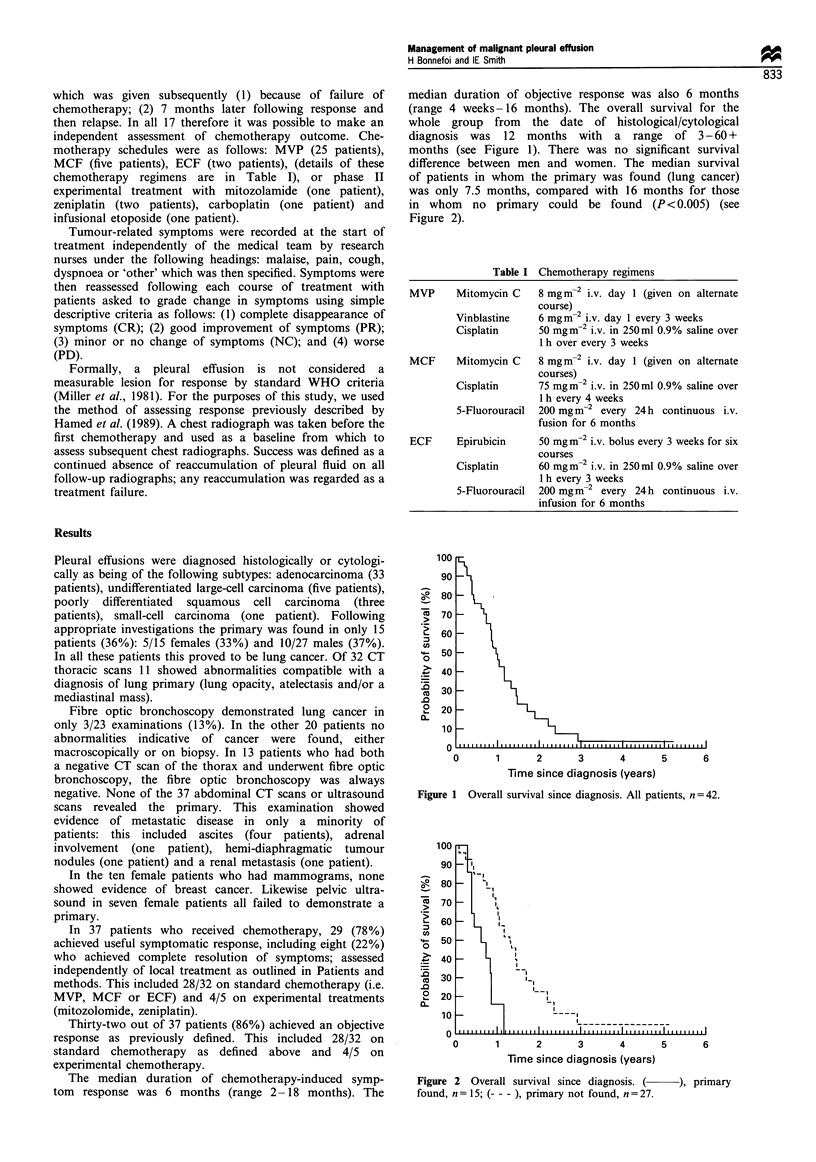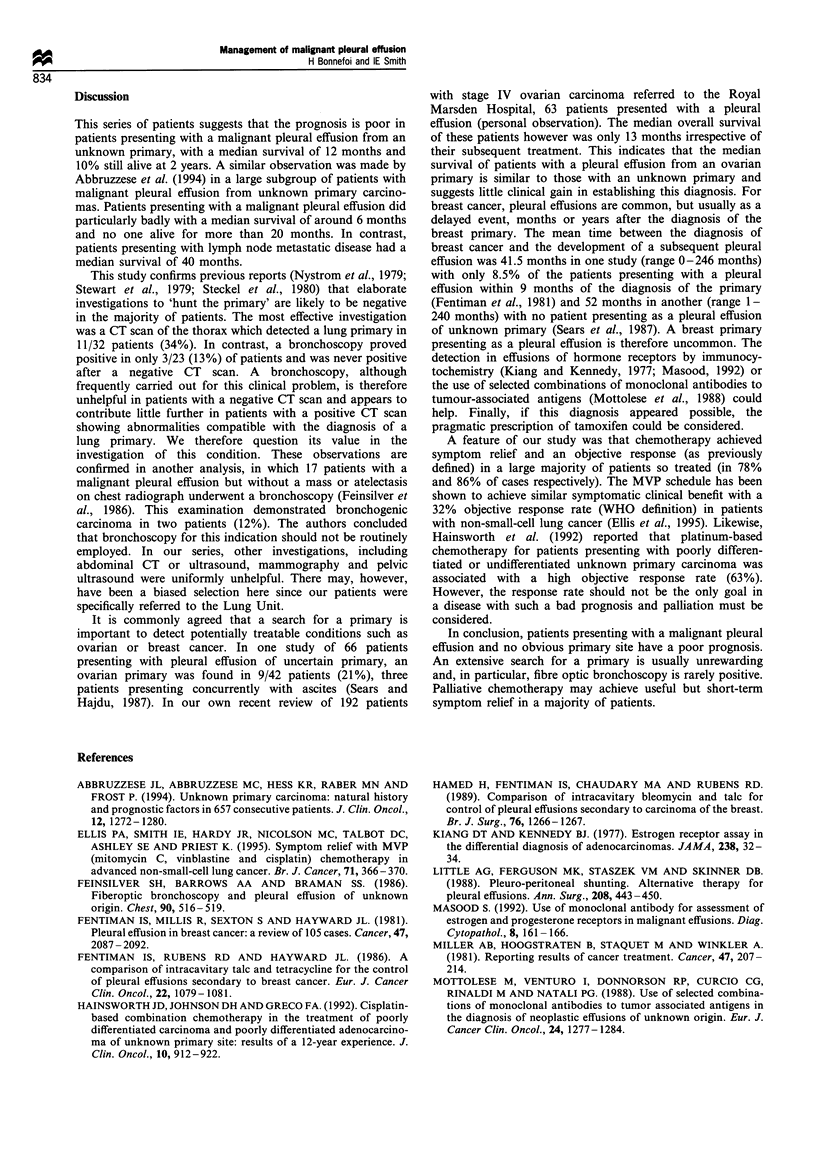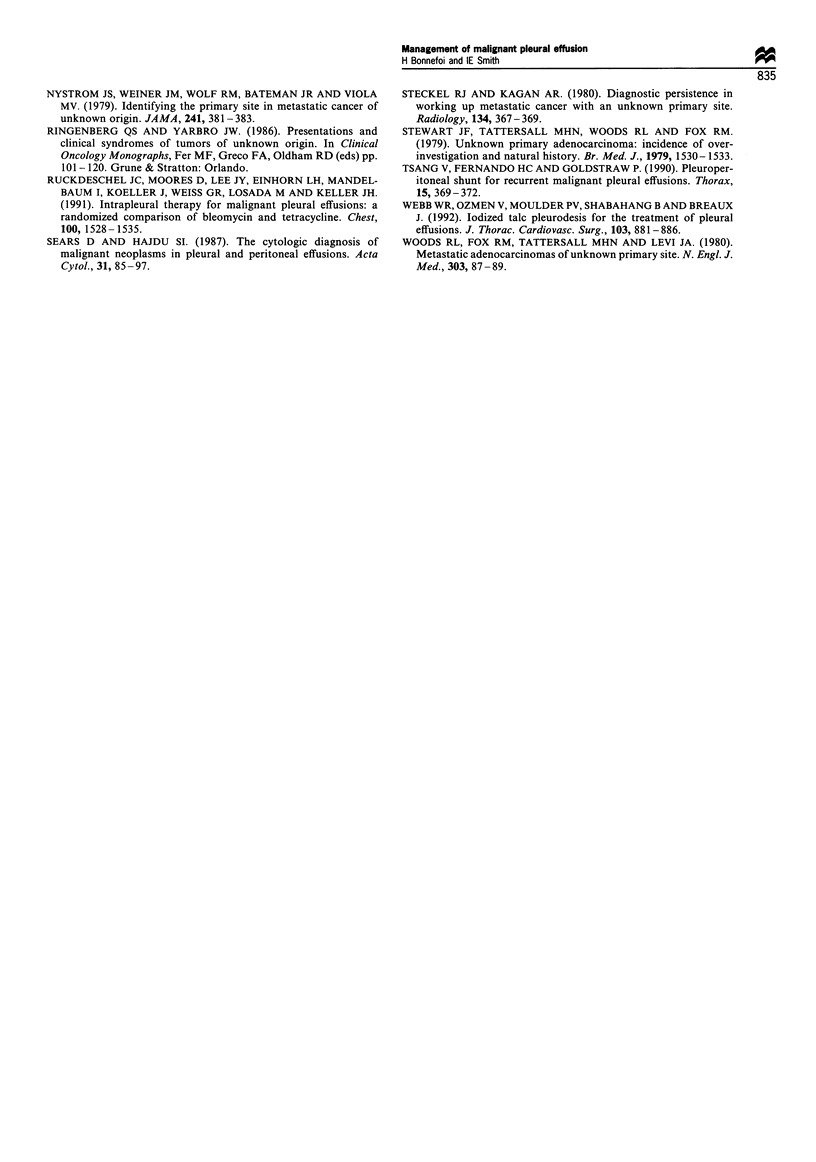# How should cancer presenting as a malignant pleural effusion be managed?

**DOI:** 10.1038/bjc.1996.444

**Published:** 1996-09

**Authors:** H. Bonnefoi, I. E. Smith

**Affiliations:** Lung Unit, Royal Marsden NHS Trust, Sutton, Surrey, UK.

## Abstract

The objective of the study was to review the natural history of patients with a malignant pleural effusion but without obvious evidence of a primary, to assess the value of investigations used to look for a primary and to assess the response to palliative chemotherapy. This was done by a retrospective study of patients' notes at the Lung Unit, Royal Marsden Hospital, Sutton, Surrey. Improvement in tumour-related symptoms (and duration) on chemotherapy was assessed by the patient before the first course of chemotherapy and following each course using simple descriptive criteria as follows: (1) complete disappearance of symptoms (CR); (2) good improvement in symptoms (PR); (3) minor or no change in symptoms (NC); (4) worse symptoms (PD). Pleural effusion objective response (and duration) according to Hamed definition: success defined as a continued absence of reaccumulation of pleural fluid on all follow-up radiographs; any reaccumulation was regarded as a treatment failure. Overall survival was measured from the date of histological/cytological diagnosis to death. The study included 42 patients, 27 males and 15 females with a median age of 55 years. A primary was found in 15 patients (36%), and considered to be lung cancer. A total of 11/32 (34%) had a thoracic computed tomography (CT) scan with abnormalities compatible with a diagnosis of lung primary. When thoracic CT scan was negative, fibre optic bronchoscopy was always negative (0/13). Abdominal and pelvic CT scan, abdominal ultrasound, pelvic ultrasound and mammograms failed to reveal the primary. Twenty-three patients underwent local treatment and 37 received systemic chemotherapy. A total of 29/37 (78%) patients achieved symptomatic improvement (median duration, 6 months) and 32/37 (86%) an objective response of their pleural effusion on chemotherapy (median duration, 6 months). The median survival of the whole group was 12 months (3-60+ months). In this series the thoracic CT led to a diagnosis of lung primary in 34% of the cases. Other radiological examinations and bronchoscopy were unhelpful. Chemotherapy achieved symptom relief in 78% of patients.


British Journal of Cancer (1996) 74, 832-835
© 1996 Stockton Press All rights reserved 0007-0920/96 $12.00

### How should cancer presenting as a malignant pleural effusion be managed?

H Bonnefoi and IE Smith

*Lung Unit and Section of Medicine, The Royal Marsden NHS Trust, Downs Road, Sutton, Surrey SM2 5PT, UK.*

**Summary** The objective of the study was to review the natural history of patients with a malignant pleural effusion but without obvious evidence of a primary, to assess the value of investigations used to look for a primary and to assess the response to palliative chemotherapy. This was done by a retrospective study of patients' notes at the Lung Unit, Royal Marsden Hospital, Sutton, Surrey. Improvement in tumour-related symptoms (and duration) on chemotherapy was assessed by the patient before the first course of chemotherapy and following each course using simple descriptive criteria as follows: (1) complete disappearance of symptoms (CR); (2) good improvement in symptoms (PR); (3) minor or no change in symptoms (NC); (4) worse symptoms (PD). Pleural effusion objective response (and duration) according to Hamed definition: success defined as a continued absence of reaccumulation of pleural fluid on all follow-up radiographs; any reaccumulation was regarded as a treatment failure. Overall survival was measured from the date of histological/cytological diagnosis to death. The study included 42 patients, 27 males and 15 females with a median age of 55 years. A primary was found in 15 patients (36%), and considered to be lung cancer. A total of 11/32 (34%) had a thoracic computed tomography (CT) scan with abnormalities compatible with a diagnosis of lung primary. When thoracic CT scan was negative, fibre optic bronchoscopy was always negative (0/13). Abdominal and pelvic CT scan, abdominal ultrasound, pelvic ultrasound and mammograms failed to reveal the primary. Twenty-three patients underwent local treatment and 37 received systemic chemotherapy. A total of 29/37 (78%) patients achieved symptomatic improvement (median duration, 6 months) and 32/37 (86%) an objective response of their pleural effusion on chemotherapy (median duration, 6 months). The median survival of the whole group was 12 months (3-60+ months). In this series the thoracic CT led to a diagnosis of lung primary in 34% of the cases. Other radiological examinations and bronchoscopy were unhelpful. Chemotherapy achieved symptom relief in 78% of patients.

**Keywords:** malignant pleural effusion; unknown primary carcinoma

Malignant pleural effusion from an unknown primary is a common presenting problem in cancer medicine and is reported as accounting for 12% of all presentations of unknown primary carcinoma (Abbruzzese *et al.*, 1994). Moreover, between 6 and 15% of all malignant pleural effusions are from an unknown primary (Ringenberg and Yarbro, 1986; Sears and Hajdu, 1987).

Several studies have assessed the value of an extensive series of 'hunt the primary' investigations in patients with carcinoma of unknown primary affecting different sites (Nystrom *et al.*, 1979; Stewart *et al.*, 1979; Steckel and Kagan, 1980), but for patients presenting with a malignant pleural effusion of uncertain primary, there is no clearly defined management policy (Ringenberg and Yarbro, 1986).

Likewise there is no clearly defined policy for treatment. Most published data are about local treatment: these include instillation of intrapleural sclerosing agents such as bleomycin (Hamed *et al.*, 1989; Ruckdeschel *et al.*, 1991), tetracycline (Fentiman *et al.*, 1986; Ruckdeschel *et al.*, 1991), talc (Hamed *et al.*, 1989; Fentiman *et al.*, 1986), iodised talc (Webb *et al.*, 1992) and, more recently, pleuroperitoneal shunts (Little *et al.*, 1988; Tsang *et al.*, 1990). For systemic chemotherapy for carcinomas of unknown primary, the survival impact has been assessed in a few studies (Woods *et al.*, 1980; Hainsworth *et al.*, 1992; Abbruzzese *et al.*, 1994), but not specifically for pleural effusions; moreover we are unaware of any published attempt to assess the symptomatic benefit of this approach.

We have therefore retrospectively reviewed 42 consecutive patients referred to the Royal Marsden Hospital with a malignant pleural effusion as the presenting clinical problem but without obvious evidence of a primary. We have assessed the value of the investigations used to look for a primary and reviewed the natural history and response to palliative chemotherapy.

Correspondence: IE Smith
Received 8 December 1995; revised 21 March 1996; accepted 22 March 1996

### Patients and methods

The clinical records of 42 consecutive patients referred to the Lung Unit at the Royal Marsden Hospital from 1985 to 1994 with a malignant pleural effusion as the presenting clinical problem but without evidence of a primary were retrospectively reviewed. Patients with pleural effusion as an incidental finding to other overt clinical or radiological features of cancer were excluded (e.g. in association with previously established breast or lung cancer).

Of these patients 27 were males and 15 females with a median age of 55 years (range 33-74 years). WHO performance status was as follows: PS 0, five patients; PS 1, 26 patients; PS 2, ten patients; PS 3, one patient.

All patients had detailed history and clinical examination with particular emphasis on examination of breasts, prostate and testes. All patients also had a standard full blood count, serum liver function tests and chest radiograph. In addition, the following further investigations were carried out: CT scan of thorax (32 patients), fibre optic bronchoscopy (23 patients), abdominal CT scan or ultrasound (37 patients), mammography (ten patients) and pelvic ultrasound (ten female patients).

Twenty-three patients received local treatment including pleural aspiration to dryness with intrapleural bleomycin (seven patients), tetracycline (ten patients), talc (four patients), pleuroperitoneal shunts (two patients, after intrapleural tetracycline in one) and surgical pleural decortification (one patient). Thirty-seven patients received chemotherapy. In 17 of these, this was after local treatment in patients with persisting or progressive symptoms. In two patients this was concurrent with local treatment but for symptoms (pain and cough) considered independent of the effusion. In two patients this was before local treatment which was given subsequently (1) because of failure of chemotherapy; (2) 7 months later following response and then relapse. In all 17 therefore it was possible to make an independent assessment of chemotherapy outcome. Chemotherapy schedules were as follows: MVP (25 patients), MCF (five patients), ECF (two patients), (details of these chemotherapy regimens are in Table I), or phase II experimental treatment with mitozolamide (one patient), zeniplatin (two patients), carboplatin (one patient) and infusional etoposide (one patient).

Tumour-related symptoms were recorded at the start of treatment independently of the medical team by research nurses under the following headings: malaise, pain, cough, dyspnoea or 'other' which was then specified. Symptoms were then reassessed following each course of treatment with patients asked to grade change in symptoms using simple descriptive criteria as follows: (1) complete disappearance of symptoms (CR); (2) good improvement of symptoms (PR); (3) minor or no change of symptoms (NC); and (4) worse (PD).

Formally, a pleural effusion is not considered a measurable lesion for response by standard WHO criteria (Miller *et al.*, 1981). For the purposes of this study, we used the method of assessing response previously described by Hamed *et al.* (1989). A chest radiograph was taken before the first chemotherapy and used as a baseline from which to assess subsequent chest radiographs. Success was defined as a continued absence of reaccumulation of pleural fluid on all follow-up radiographs; any reaccumulation was regarded as a treatment failure.

### Results

Pleural effusions were diagnosed histologically or cytologically as being of the following subtypes: adenocarcinoma (33 patients), undifferentiated large-cell carcinoma (five patients), poorly differentiated squamous cell carcinoma (three patients), small-cell carcinoma (one patient). Following appropriate investigations the primary was found in only 15 patients (36%): 5/15 females (33%) and 10/27 males (37%). In all these patients this proved to be lung cancer. Of 32 CT thoracic scans 11 showed abnormalities compatible with a diagnosis of lung primary (lung opacity, atelectasis and/or a mediastinal mass).

Fibre optic bronchoscopy demonstrated lung cancer in only 3/23 examinations (13%). In the other 20 patients no abnormalities indicative of cancer were found, either macroscopically or on biopsy. In 13 patients who had both a negative CT scan of the thorax and underwent fibre optic bronchoscopy, the fibre optic bronchoscopy was always negative. None of the 37 abdominal CT scans or ultrasound scans revealed the primary. This examination showed evidence of metastatic disease in only a minority of patients: this included ascites (four patients), adrenal involvement (one patient), hemi-diaphragmatic tumour nodules (one patient) and a renal metastasis (one patient).

In the ten female patients who had mammograms, none showed evidence of breast cancer. Likewise pelvic ultrasound in seven female patients all failed to demonstrate a primary.

In 37 patients who received chemotherapy, 29 (78%) achieved useful symptomatic response, including eight (22%) who achieved complete resolution of symptoms; assessed independently of local treatment as outlined in Patients and methods. This included 28/32 on standard chemotherapy (i.e. MVP, MCF or ECF) and 4/5 on experimental treatments (mitozolomide, zeniplatin).

Thirty-two out of 37 patients (86%) achieved an objective response as previously defined. This included 28/32 on standard chemotherapy as defined above and 4/5 on experimental chemotherapy.

The median duration of chemotherapy-induced symptom response was 6 months (range 2–18 months). The median duration of objective response was also 6 months (range 4 weeks–16 months). The overall survival for the whole group from the date of histological/cytological diagnosis was 12 months with a range of 3–60+ months (see Figure 1). There was no significant survival difference between men and women. The median survival of patients in whom the primary was found (lung cancer) was only 7.5 months, compared with 16 months for those in whom no primary could be found ($P<0.005$) (see Figure 2).

**Table I** Chemotherapy regimens

| | | |
|---|---|---|
| MVP | Mitomycin C | 8 mg m$^{-2}$ i.v. day 1 (given on alternate course) |
| | Vinblastine | 6 mg m$^{-2}$ i.v. day 1 every 3 weeks |
| | Cisplatin | 50 mg m$^{-2}$ i.v. in 250 ml 0.9% saline over 1 h over every 3 weeks |
| MCF | Mitomycin C | 8 mg m$^{-2}$ i.v. day 1 (given on alternate courses) |
| | Cisplatin | 75 mg m$^{-2}$ i.v. in 250 ml 0.9% saline over 1 h every 4 weeks |
| | 5-Fluorouracil | 200 mg m$^{-2}$ every 24 h continuous i.v. fusion for 6 months |
| ECF | Epirubicin | 50 mg m$^{-2}$ i.v. bolus every 3 weeks for six courses |
| | Cisplatin | 60 mg m$^{-2}$ i.v. in 250 ml 0.9% saline over 1 h every 3 weeks |
| | 5-Fluorouracil | 200 mg m$^{-2}$ every 24 h continuous i.v. infusion for 6 months |

**Figure 1** Overall survival since diagnosis. All patients, $n=42$.

**Figure 2** Overall survival since diagnosis. (———), primary found, $n=15$; (- - - ), primary not found, $n=27$.

### Discussion

This series of patients suggests that the prognosis is poor in patients presenting with a malignant pleural effusion from an unknown primary, with a median survival of 12 months and 10% still alive at 2 years. A similar observation was made by Abbruzzese *et al.* (1994) in a large subgroup of patients with malignant pleural effusion from unknown primary carcinomas. Patients presenting with a malignant pleural effusion did particularly badly with a median survival of around 6 months and no one alive for more than 20 months. In contrast, patients presenting with lymph node metastatic disease had a median survival of 40 months.

This study confirms previous reports (Nystrom *et al.*, 1979; Stewart *et al.*, 1979; Steckel *et al.*, 1980) that elaborate investigations to 'hunt the primary' are likely to be negative in the majority of patients. The most effective investigation was a CT scan of the thorax which detected a lung primary in 11/32 patients (34%). In contrast, a bronchoscopy proved positive in only 3/23 (13%) of patients and was never positive after a negative CT scan. A bronchoscopy, although frequently carried out for this clinical problem, is therefore unhelpful in patients with a negative CT scan and appears to contribute little further in patients with a positive CT scan showing abnormalities compatible with the diagnosis of a lung primary. We therefore question its value in the investigation of this condition. These observations are confirmed in another analysis, in which 17 patients with a malignant pleural effusion but without a mass or atelectasis on chest radiograph underwent a bronchoscopy (Feinsilver *et al.*, 1986). This examination demonstrated bronchogenic carcinoma in two patients (12%). The authors concluded that bronchoscopy for this indication should not be routinely employed. In our series, other investigations, including abdominal CT or ultrasound, mammography and pelvic ultrasound were uniformly unhelpful. There may, however, have been a biased selection here since our patients were specifically referred to the Lung Unit.

It is commonly agreed that a search for a primary is important to detect potentially treatable conditions such as ovarian or breast cancer. In one study of 66 patients presenting with pleural effusion of uncertain primary, an ovarian primary was found in 9/42 patients (21%), three patients presenting concurrently with ascites (Sears and Hajdu, 1987). In our own recent review of 192 patients with stage IV ovarian carcinoma referred to the Royal Marsden Hospital, 63 patients presented with a pleural effusion (personal observation). The median overall survival of these patients however was only 13 months irrespective of their subsequent treatment. This indicates that the median survival of patients with a pleural effusion from an ovarian primary is similar to those with an unknown primary and suggests little clinical gain in establishing this diagnosis. For breast cancer, pleural effusions are common, but usually as a delayed event, months or years after the diagnosis of the breast primary. The mean time between the diagnosis of breast cancer and the development of a subsequent pleural effusion was 41.5 months in one study (range 0–246 months) with only 8.5% of the patients presenting with a pleural effusion within 9 months of the diagnosis of the primary (Fentiman *et al.*, 1981) and 52 months in another (range 1–240 months) with no patient presenting as a pleural effusion of unknown primary (Sears *et al.*, 1987). A breast primary presenting as a pleural effusion is therefore uncommon. The detection in effusions of hormone receptors by immunocytochemistry (Kiang and Kennedy, 1977; Masood, 1992) or the use of selected combinations of monoclonal antibodies to tumour-associated antigens (Mottolese *et al.*, 1988) could help. Finally, if this diagnosis appeared possible, the pragmatic prescription of tamoxifen could be considered.

A feature of our study was that chemotherapy achieved symptom relief and an objective response (as previously defined) in a large majority of patients so treated (in 78% and 86% of cases respectively). The MVP schedule has been shown to achieve similar symptomatic clinical benefit with a 32% objective response rate (WHO definition) in patients with non-small-cell lung cancer (Ellis *et al.*, 1995). Likewise, Hainsworth *et al.* (1992) reported that platinum-based chemotherapy for patients presenting with poorly differentiated or undifferentiated unknown primary carcinoma was associated with a high objective response rate (63%). However, the response rate should not be the only goal in a disease with such a bad prognosis and palliation must be considered.

In conclusion, patients presenting with a malignant pleural effusion and no obvious primary site have a poor prognosis. An extensive search for a primary is usually unrewarding and, in particular, fibre optic bronchoscopy is rarely positive. Palliative chemotherapy may achieve useful but short-term symptom relief in a majority of patients.

### References

ABBRUZZESE JL, ABBRUZZESE MC, HESS KR, RABER MN AND FROST P. (1994). Unknown primary carcinoma: natural history and prognostic factors in 657 consecutive patients. *J. Clin. Oncol.*, **12**, 1272–1280.

ELLIS PA, SMITH IE, HARDY JR, NICOLSON MC, TALBOT DC, ASHLEY SE AND PRIEST K. (1995). Symptom relief with MVP (mitomycin C, vinblastine and cisplatin) chemotherapy in advanced non-small-cell lung cancer. *Br. J. Cancer*, **71**, 366–370.

FEINSILVER SH, BARROWS AA AND BRAMAN SS. (1986). Fiberoptic bronchoscopy and pleural effusion of unknown origin. *Chest*, **90**, 516–519.

FENTIMAN IS, MILLIS R, SEXTON S AND HAYWARD JL. (1981). Pleural effusion in breast cancer: a review of 105 cases. *Cancer*, **47**, 2087–2092.

FENTIMAN IS, RUBENS RD AND HAYWARD JL. (1986). A comparison of intracavitary talc and tetracycline for the control of pleural effusions secondary to breast cancer. *Eur. J. Cancer Clin. Oncol.*, **22**, 1079–1081.

HAINSWORTH JD, JOHNSON DH AND GRECO FA. (1992). Cisplatin-based combination chemotherapy in the treatment of poorly differentiated carcinoma and poorly differentiated adenocarcinoma of unknown primary site: results of a 12-year experience. *J. Clin. Oncol.*, **10**, 912–922.

HAMED H, FENTIMAN IS, CHAUDARY MA AND RUBENS RD. (1989). Comparison of intracavitary bleomycin and talc for control of pleural effusions secondary to carcinoma of the breast. *Br. J. Surg.*, **76**, 1266–1267.

KIANG DT AND KENNEDY BJ. (1977). Estrogen receptor assay in the differential diagnosis of adenocarcinomas. *JAMA*, **238**, 32–34.

LITTLE AG, FERGUSON MK, STASZEK VM AND SKINNER DB. (1988). Pleuro-peritoneal shunting. Alternative therapy for pleural effusions. *Ann. Surg.*, **208**, 443–450.

MASOOD S. (1992). Use of monoclonal antibody for assessment of estrogen and progesterone receptors in malignant effusions. *Diag. Cytopathol.*, **8**, 161–166.

MILLER AB, HOOGSTRATEN B, STAQUET M AND WINKLER A. (1981). Reporting results of cancer treatment. *Cancer*, **47**, 207–214.

MOTTOLESE M, VENTURO I, DONNORSON RP, CURCIO CG, RINALDI M AND NATALI PG. (1988). Use of selected combinations of monoclonal antibodies to tumor associated antigens in the diagnosis of neoplastic effusions of unknown origin. *Eur. J. Cancer Clin. Oncol.*, **24**, 1277–1284.

NYSTROM JS, WEINER JM, WOLF RM, BATEMAN JR AND VIOLA MV. (1979). Identifying the primary site in metastatic cancer of unknown origin. *JAMA*, **241**, 381–383.

RINGENBERG QS AND YARBRO JW. (1986). Presentations and clinical syndromes of tumors of unknown origin. In *Clinical Oncology Monographs*, Fer MF, Greco FA, Oldham RD (eds) pp. 101–120. Grune & Stratton: Orlando.

RUCKDESCHEL JC, MOORES D, LEE JY, EINHORN LH, MANDELBAUM I, KOELLER J, WEISS GR, LOSADA M AND KELLER JH. (1991). Intrapleural therapy for malignant pleural effusions: a randomized comparison of bleomycin and tetracycline. *Chest*, **100**, 1528–1535.

SEARS D AND HAJDU SI. (1987). The cytologic diagnosis of malignant neoplasms in pleural and peritoneal effusions. *Acta Cytol.*, **31**, 85–97.

STECKEL RJ AND KAGAN AR. (1980). Diagnostic persistence in working up metastatic cancer with an unknown primary site. *Radiology*, **134**, 367–369.

STEWART JF, TATTERSALL MHN, WOODS RL AND FOX RM. (1979). Unknown primary adenocarcinoma: incidence of over-investigation and natural history. *Br. Med. J.*, **1979**, 1530–1533.

TSANG V, FERNANDO HC AND GOLDSTRAW P. (1990). Pleuroperitoneal shunt for recurrent malignant pleural effusions. *Thorax*, **15**, 369–372.

WEBB WR, OZMEN V, MOULDER PV, SHABAHANG B AND BREAUX J. (1992). Iodized talc pleurodesis for the treatment of pleural effusions. *J. Thorac. Cardiovasc. Surg.*, **103**, 881–886.

WOODS RL, FOX RM, TATTERSALL MHN AND LEVI JA. (1980). Metastatic adenocarcinomas of unknown primary site. *N. Engl. J. Med.*, **303**, 87–89.